# A Pilot Study Investigating the Influence of Glucagon-Like Peptide-1 Receptor Single Nucleotide Polymorphisms on Gastric Emptying Rate in Caucasian Men

**DOI:** 10.3389/fphys.2018.01331

**Published:** 2018-09-25

**Authors:** Adora M. W. Yau, John McLaughlin, Ronald J. Maughan, William Gilmore, Jason J. Ashworth, Gethin H. Evans

**Affiliations:** ^1^School of Healthcare Science, Manchester Metropolitan University, Manchester, United Kingdom; ^2^Division of Diabetes, Endocrinology and Gastroenterology, School of Medical Sciences, The University of Manchester, Manchester, United Kingdom; ^3^Salford Royal Hospitals, Salford, United Kingdom; ^4^School of Medicine, University of St Andrews, St Andrews, United Kingdom; ^5^School of Biomedical Sciences, Ulster University, Coleraine, United Kingdom

**Keywords:** GLP-1 receptor, single nucleotide polymorphisms, gastric emptying rate, GLP-1, gastrointestinal motility

## Abstract

Gastric emptying rate in humans is subject to large individual variability, but previous research on the influence of genetics is scarce. Variation in the glucagon-like peptide-1 receptor (GLP1R) gene is a plausible candidate gene to partially explain the high variance. This study aimed to investigate the influence of genetic variation in the GLP1R gene on gastric emptying rate of a glucose solution in humans. Forty eight healthy Caucasian males took part in this investigation. Gastric emptying rate of a 6% glucose solution was assessed using the 13C breath test method and a venous blood sample was obtained from each participant. Participants were genotyped for 27 Tag single nucleotide polymorphisms (SNPs) in the GLP1R locus using Sequenom MassARRAY iPLEX GOLD analysis and MALDI-TOF mass spectrometry. The time at which maximal emptying rate occurred (T_lag_) was faster in participants with the CC genotype than in TT and TC genotypes for SNP rs742764: [median (quartiles) CC, 35 (30–36) min vs. TT, 43 (39–46) min, and TC, 41 (39–45) min; *P* < 0.01]. T_lag_ was also slower in participants with the AA genotype compared to the TT and TA genotypes for SNP rs2254336: [AA, 43 (39–49) min vs. TT, 36 (34–41) min, and TA, 39 (35–42) min; *P* < 0.05]. Analysis by phenotype also showed differences in half-emptying time (T_12_) and T_lag_ for SNPs rs9283907, rs2268657, and rs2254336. Several neighboring Tag SNPs within the GLP1R gene were found to be associated with gastric emptying rate, and should be further investigated.

## Introduction

An increasing number of genetic variants within the genes coding for gastrointestinal hormones and their receptors are being found to be associated with obesity phenotypes, appetite, and food regulation. As many of the gastrointestinal hormones involved in appetite regulation also play a role in the regulation of gastric emptying, it is possible that genetic variations in gastrointestinal hormones associated with obesity may affect gastric emptying rate. However, research on the potential influence of genetics on gastric emptying rate is scarce. Acosta et al. (2014) reported a common genetic variant rs17782313 in the Melanocortin 4 receptor (MC4R) gene, which has previously been found to be strongly associated with common obesity (Loos et al., 2008; Vogel et al., 2011), to be associated with reduced gastric emptying rate and satiation. In addition, Cremonini et al. (2005) found an association between the 779T > C polymorphism in the cholecystokinin (CCK) gene and slower gastric emptying rate. On the other hand, Jones et al. (2010) found no effect of common genetic polymorphisms of the CCK or CCK-1 receptor genes on gastric emptying rate.

Genetic variation within a gene related to the action of the gastrointestinal hormone glucagon-like peptide-1 (GLP-1) presents a plausible area of investigation, as GLP-1 has been shown to influence gastric emptying rate (Wettergren et al., 1993; Wishart et al., 1998). Furthermore, large inter-individual variations in gastric emptying rate of a glucose solution as well as the large differences in GLP-1 hormone responses to carbohydrate ingestion have been observed (Yau et al., 2014, 2017). GLP-1 exerts its effects via a G-protein coupled receptor called the GLP-1 receptor (GLP1R). Stimulation of this receptor by GLP-1 triggers cAMP production as the primary signal transduction pathway (Mayo et al., 2003). The GLP1R recognizes GLP-1 specifically despite the hormone having strong sequence homology to other hormones within the glucagon-related family of peptides, and furthermore, does not bind to a number of other related peptides, including secretin (Fehmann et al., 1994).

Genetic polymorphisms of the GLP1R gene have previously been investigated in relation to insulin secretion (Sathananthan et al., 2010) and the pathogenesis of diabetes (Tanizawa et al., 1994; Tokuyama et al., 2004; Beinborn et al., 2005). Only one study has previously investigated the influence of GLP1R genetic variation on gastric emptying rate, but in mice (Kumar et al., 2008). The presence of a non-synonymous cysteine to tyrosine substitution at amino acid 416 of the GLP1R gene found in CAST strain mice was associated with reduced GLP1R expression and significantly faster (by 20%) gastric emptying rate compared to B6 strain mice. This was also seen for the congenic strains where gastric emptying rate was significantly higher in B6.CAST-17 congenic mice compared to homozygous B6 controls. Furthermore, administration of the GLP1R antagonist extendin-(9-36) resulted in no increase in gastric emptying rate compared to a 10% increase in the homozygous B6 control mice. The GLP1R gene is therefore a plausible candidate gene for a genetic association study on gastric emptying rate in humans. The human GLP1R gene consists of 13 exons interrupted by 12 introns (Wilmen et al., 1998) and is situated on chromosome 6, band p21.1 (Stoffel et al., 1993). A major transcription start point and a minor transcription start point 42 base pairs (bp) and 360 bp upstream of the translation initiation site, respectively, have been reported (Lankat-Buttgereit and Goke, 1997). Three putative Sp1 binding sites have also been located in the proximal 5′ flanking sequence at -108, -173, and -389 bp from the translation initiation codon (Lankat-Buttgereit and Goke, 1997). The receptor is 463 amino acids in length and is highly conserved between species with 90% being identical to rat GLP1R (Dillon et al., 1993) and approximately 95% homology with mice GLP1R. The primary aim of this study was to investigate the influence of genetic variation in the GLP1R gene on gastric emptying rate of a glucose solution in humans.

## Materials and Methods

### Ethical Approval

Forty eight British and South-Western European Caucasian males aged 18–35 years (mean ± SD, age 23 ± 5 years, height 178 ± 7 cm, body mass 75.8 ± 11.2 kg, body mass index 23.9 ± 3.3 kg.m^-2^, and estimated body fat percentage 19 ± 6%) volunteered for this study. All participants were non-smokers, were not taking medication with any known effect on gastrointestinal function, and had no known history of chronic gastrointestinal disease or other medical conditions as assessed by a medical screening questionnaire. All participants provided written informed consent prior to participation. The study was granted prior ethical approval from the Manchester Metropolitan University Ethical Advisory committee and was performed in accordance with the Declaration of Helsinki (2008).

### Experimental Trial

All participants attended the laboratory for one experimental trial. In the 24 h preceding the trial, participants were requested to refrain from alcohol and caffeine consumption, and the performance of strenuous physical activity. Participants arrived at the laboratory in the morning following an overnight fast from 2100 h, with the exception of drinking 500 mL of water 90 min prior to arrival at the laboratory, in an attempt to ensure a consistent and adequate hydration status.

Upon arrival at the laboratory, participants were asked to completely empty their bladder. Body mass was then recorded. Following this, a whole blood sample was obtained by venipuncture of an antecubital vein. Once this procedure was completed, participants ingested 595 mL of a 6% glucose solution as quickly as they were comfortably able over a maximum period of 2 min. Test solutions were prepared fresh on the morning of the trial containing 39.6 g glucose monohydrate ^1^ dissolved in water and made up to a volume of 600 mL. In addition, solutions contained 100 mg of ^13^C sodium acetate (Cambridge Isotopes Laboratories, Inc., Andover, MA, United States) for the assessment of gastric emptying rate. The drink was served at ambient temperature and a 5 mL sample retained for later analysis of osmolality. Participants remained seated throughout a 60 min post-ingestion sampling period where end expiratory breath samples were collected at baseline then every 10 min after ingestion into foil bags (Wagner Analyzen-Technik, Breman, Germany) by exhalation through a mouthpiece. Bags were then sealed with a plastic stopper and stored for later analysis. Breath samples were analyzed for the ratio of ^13^CO_2_ to ^12^CO_2_ by non-dispersive infrared spectroscopy, with the difference in the ratio from baseline to post-breath samples expressed as delta over baseline (DOB). Half emptying time (T_½_) and the time at which maximal emptying rate occurred (T_lag_) were calculated using the manufacturer’s integrated software evaluation embedded with equations previously described (Ghoos et al., 1993). Each participant’s own physiological CO_2_ production assumed as 300 mmol CO_2_ per m^2^ body surface per hour was set as default and body surface area was calculated by the integrated software according to a previously described formula (Haycock et al., 1978). Following all sample collections at 60 min, participants were free to leave the laboratory.

### Genotyping

Genomic DNA was extracted from 3 mL of whole blood using Flexigene DNA Kit (Qiagen, West Sussex, United Kingdom), according to the manufacturer’s instructions, except for the final step of resuspension in 300 μL of nuclease-free ultrapure water. Extracted DNA was subsequently quantified using a NanoDrop 2000 (Thermo Scientific, 116 Loughborough, United Kingdom) and normalized to a concentration of 40 ng/μL then stored at -20°C before working concentrations of 20 ng/μL were prepared in 96-well plate format prior to use.

Twenty-eight tag single nucleotide polymorphisms (SNPs) in the GLP1R locus incorporating 10,000 bp upstream and downstream of the major transcription initiation site and the last exon, respectively (Chr6: 39114595….39173498) were selected from HapMap^2^ (HapMap Data release 27/phase II + III, Feb09, on NCBI B36 assembly, dbSNP b126). The Tagger algorithm for multi-marker tagging with *r*^2^ > 0.8 and minor allele frequency (MAF) > 0.1 was used (de Bakker et al., 2005). Furthermore, three additional non-synonymous SNPs were selected based on previous literature. One of two SNPs found to be associated with insulin secretion in response to exogenous infusion of GLP-1 by Sathananthan et al. (2010), which was not already in the generated list of 28 tag SNPs was genotyped. The other two additional SNPs genotyped were two in very close proximity (two amino acids upstream and three amino acids downstream) to the equivalent locus of the SNP found in mice to be associated with gastric emptying rate (Kumar et al., 2008). All 31 SNPs were genotyped using Sequenom MassARRAY iPLEX GOLD analysis. Forward and reverse primers as well as extension probes were designed using Sequenom Assay Design Suite (v1.0) which produced two appropriate assay plexes; plex 1 containing 24 SNPs and plex 2 containing seven SNPs (**Table 1**). Primers were purchased from Metabion International AG (Martinsreid, Germany).

**Table 1:** List of SNPs analyzed and primers used.

	SNP ID	Pos. from major transcription site (bp)	Forward primer	Reverse primer	Extension probe
1	rs7738586	-9,033	ACGTTGGATGACACCCAGACTGACGTTATC	ACGTTGGATGTTGTGCAAAAGCAGCCCAAG	TCAAAGTGATTGTCACCATAAG
2	rs9380825	-5,610	ACGTTGGATGTGAGCCAGGAAGTGATGTTC	ACGTTGGATGTCTCATCCAGGCAGCAAGTA	cccCTGCACCAGAGCCCCT
3	rs9296274	-1,455	ACGTTGGATGTTTGGATATCGTGGCTGGAG	ACGTTGGATGTGCCTGGGTCTTCTAGCTTC	gggtGTGGAAATCTTGGACCA
4	rs926674	2,930	ACGTTGGATGACTCACACATACCTGGGAAC	ACGTTGGATGATACAGACAGGTAGTCTGAG	tagcAGGGAGTAGGCTATATGA
5	rs2268657	3,969	ACGTTGGATGGTTCTGCCGTCCATAAAATG	ACGTTGGATGTACAGGGCTTGAGAAGTCAC	ggaagTGGGCATATCATTCTTCTCA
6	rs13202369	6,125	ACGTTGGATGTGATCCACCAGGACTTGCTC	ACGTTGGATGTAACAGCTGCAAAGGTGTTG	gacaTCCTCAGCTGTGGCTAAT
7	rs3799707	6,937	ACGTTGGATGTTTGGTTGCTGTGTCAGAGG	ACGTTGGATGGCACTCACTTACAGATGCAC	AGATGCACTCAACACA(Inosine)C
8	rs10305432	7,057	ACGTTGGATGTCTTTGTAGCCCTGAACGCC	ACGTTGGATGAGTCCTTCAGATCAGTGACC	CCGCACACCTTGCGA
9	rs9283907	10,130	ACGTTGGATGGCTCCTATCATCACACCTTG	ACGTTGGATGCCAGAGCATAACCTCATGCC	aagaA(Inosine)CAACTGGCCCAGAA
10	rs742764	13,261	ACGTTGGATGTCTCAGCTTCTGCATCTGTA	ACGTTGGATGTGTGGAGAGCTGCTCATGAA	ggagcGCTGCTCATGAATCCATTA
11	rs2254336	16,262	ACGTTGGATGCTGGTCTAAAAGGAGTACAC	ACGTTGGATGAAGGTAGGAGCTGGGTATTC	ggAGGCTGCATACGACCA
12	rs910163	16,847	ACGTTGGATGTGAGCCTCAGCCCAGAATAG	ACGTTGGATGCAGGGATAGCCCTCAGAATG	tATGGGGAGGAAGGGG
13	rs3765467^∗^	17,022	ACGTTGGATGAACCCCGCCTCAACTCACTC	ACGTTGGATGTGCAGAAGGACAACTCCAGC	AACTCACTCT(Inosine)TCCCCT
14	rs6923761	17,499	ACGTTGGATGTTCTCTGCTCTGGTTATCGC	ACGTTGGATGGAGTTAGGATGAAGCAGCCC	GGGCCACCTTACCTGAAGC
15	rs7766663	19,209	ACGTTGGATGAGCAATAGGTCTGCATGTGG	ACGTTGGATGCTGGTCTCCAATCTCTGGC	CCTAGCTAATTGAGAGGC
16	rs932443	25,761	ACGTTGGATGGTGAATGAAGGAGTGGCAAG	ACGTTGGATGTCCTCCCTAATCTGCCATTG	ggattGTGTGGAACAGGAAAACTC
17	rs2268646	26,943	ACGTTGGATGCCAACTGTGTCAGAGTCCTA	ACGTTGGATGGTGATGCCAGGAGGCCTTG	cccaTTCCACTTGCACATGAA
18	rs2300614	32,400	ACGTTGGATGTCCAAACCTAGGGCAGGTTC	ACGTTGGATGCCCTGCTAAAATTCTTATTTC	TCTTTCTGATCTTCAGTGTT
19	rs2268641	33,693	ACGTTGGATGCTGGGTCCTCTAAGACCTGT	ACGTTGGATGCAAAGAGTGGCCCATAAATG	ggggAAGACCTGTCCCAGGA
20	rs2268640	33,811	ACGTTGGATGTGCACTTCCTCGTTTGCATC	ACGTTGGATGTCCTCCTCCACTGCCATATC	CACTGCCATATCCTCAAAATGA
21	rs2268639	34,049	ACGTTGGATGGATAGAGAAGTGAGAAACGG	ACGTTGGATGATGAGGAGCAGAGGCCTGTA	ggcaCTGCTGCCACCTTGTCATCT
22	rs2206942	34,866	ACGTTGGATGAATTGGGAAGCTCATTCACC	ACGTTGGATGGGCAAGTCATTTTGCCTCCC	ggcGCTCATTCACCTTCATTTAC
23	rs2894420	36,523	ACGTTGGATGAACAGGGATCCTGGCTGAC	ACGTTGGATGAGTGAGGGCTTCTCAACTG	CACTGCAGTGTCTCTCT
24	rs199796313†	37,124	ACGTTGGATGTCCTTTTCCCATGGAAGGTC	ACGTTGGATGTGGATGTGCAAGTGCTCAAG	GGTCCAGCTGGAATTT
25	rs200691429†	37,140	ACGTTGGATGAAGGTCCAGCTGGAATTTCG	ACGTTGGATGTGGATGTGCAAGTGCTCAAG	gGGAAGAGCTGGGAGC
26	rs4714211	39,117	ACGTTGGATGAAGGCACCCCTTATTTGCTG	ACGTTGGATGACTTGCACCAGCACTGTTTC	ccccTTTGCTGTCTCTTCGT
27	rs10305525	39,577	ACGTTGGATGAATGGCACTGCACTCTTTCC	ACGTTGGATGCATTGCATTCAATAGTTCCC	aATTCAATAGTTCCCAGACCT
28	rs9296291	40,356	ACGTTGGATGGATGGTGAAAGTGTCATCTC	ACGTTGGATGAAGACAAGGATGAATGAAG	gTGAATGAAGTACCAGTGT
29	rs9968886	43,905	ACGTTGGATGACAGTGAGGTTTTCCCCATC	ACGTTGGATGTCATGTAGTCCAGCTTGTGC	GCTTGTGCTGCTAGTT
30	rs2143733	44,923	ACGTTGGATGCATCTAATCGATGGGTAGC	ACGTTGGATGCAGAACCCTTCTGAACCTTC	GAAATTGAATTTACAGCTTTAATAAA
31	rs9296292	46,417	ACGTTGGATGTCACAATATGTTTGGCACTG	ACGTTGGATGGCTTTGTTTTGCAGAGCTTG	TGGCACTGCCAAACT

*Unshaded SNPs analyzed in plex 1, shaded SNPs in plex 2. ^∗^Additional missense SNP associated with insulin secretion in response to exogenous infusion of GLP-1 by Sathananthan et al. (2010). †Additional missense SNPs in close proximity to equivalent SNP associated with gastric emptying rate in mice (Kumar et al., 2008)*.

Briefly, an initial locus-specific amplification was performed using polymerase chain reaction (PCR; GeneAmp PCR System 9700, Applied Biosystems) carried out in a total volume of 5 μL containing 40 ng of DNA and final concentrations of 1.25x buffer, 1.0 mM MgCl_2_, 500 μM dNTP mix, 100 nM of each forward and reverse primers, and 0.1 U/μL Hotstart *Taq.* PCR conditions consisted of an initial denaturing step at 94°C for 5 min, followed by 40 cycles of denaturing at 94°C for 20 s, annealing at 56°C for 30 s and extension at 72°C for 1 min, and then a final extension step at 72°C for 3 min. Unincorporated dNTPs within the PCR products were dephosphorylated by incubation for 40 min at 37°C followed by 5 min at 85°C with 2 μL Shrimp Alkaline Phosphatase (SAP) mixture composed of 1.7 μL 1x hME buffer and 0.3 μL SAP 1.7 U/μL. A single base extension step was then performed with the addition of 2 μL reaction mix containing 50 μM of each ddNTP, 3.3:6.6 μM extension probe from low:high mass (2.8:5.6 μM for plex 2), 1.0x buffer, and 1.25 U Thermo Sequenase. Probe extension conditions consisted of a two-step 200 short cycle program involving initial denaturing at 94°C for 30 s, 40 cycles of denaturing at 94°C for 5 s, annealing at 52°C for 5 s, and extension at 80°C for 5 s where the annealing and extension steps also cycled five times, before a final extension at 72°C for 3 min. Resulting products were diluted with 20 μL nuclease-free ultrapure water and desalted with 6 mg resin by gentle inversion for 10 min before centrifugation at 4000 rpm for 5 min. Products were subsequently nano-dispensed (Samsung Sequenom MassARRAY Nanodispenser) onto a 384-element SpectroCHIP II bioarray and analyzed by MALDI-TOF mass spectrometry.

### Data and Statistical Analysis

Genotype results were visually checked and appropriate manual genotype assignment was made upon spectrum inspection where no automated genotype calling had successfully taken place. Differences in gastric emptying T_½_ and T_lag_ were examined between genotype and phenotype groups. For each SNP analyzed, it was hypothesized that there was a significant difference in gastric emptying rate between genotype groups or phenotype groups. Normality tests indicated that the majority of data were not normally distributed. Furthermore, as group sizes were unequal, non-parametric statistical analysis comprising Kruskal–Wallace and Mann–Whitney *U*-tests were utilized accordingly. Where appropriate, *post hoc* tests of Mann–Whitney *U* with Bonferroni correction were applied. All data were analyzed using SPSS Statistics for Windows (IBM, New York, NY, United States). Statistical significance was accepted at the 5% level and results presented as median and quartiles unless stated otherwise.

## Results

### SNP Genotyping

Twenty-seven of the 31 SNPs were successfully analyzed for variants. No variants occurred among the participants for three SNPs, Tag SNP 3 within the promoter region (rs9296274) and two of the three selected missense polymorphism, SNPs 24 (rs199796313) and 25 (rs200691429). Genotyping failed in all participants for SNP 13 (rs3765467), one of the three additional missense SNPs. The occasional failure to successfully genotype one participant occurred in five SNPs, reducing the total participant number (*n*) to 47 instead of 48. Frequencies of each genotype are shown in **Table 2**.

**Table 2:** SNP genotype frequencies.

		Genotype						
	SNP ID	Homozygous minor allele		Heterozygous		Homozygous major allele		
		Genotype	*n*	Genotype	*n*	Genotype	*n*	Total
1	rs7738586	AA	0	CA	10	CC	38	48
2	rs9380825	AA	5	AG	28	GG	15	48
4	rs926674	TT	3	TC	6	CC	39	48
5	rs2268657	GG	9	AG	27	AA	11	47
6	rs13202369	GG	2	AG	21	AA	25	48
7	rs3799707	TT	3	GT	19	GG	26	48
8	rs10305432	CC	3	CT	19	TT	26	48
9	rs9283907	AA	1	AG	9	GG	38	48
10	rs742764	CC	8	TC	25	TT	14	47
11	rs2254336	TT	9	TA	23	AA	16	48
12	rs910163	CC	3	TC	18	TT	27	48
14	rs6923761	AA	7	GA	28	GG	13	48
15	rs7766663	GG	10	GT	23	TT	15	48
16	rs932443	GG	5	AG	21	AA	22	48
17	rs2268646	AA	0	AG	10	GG	38	48
18	rs2300614	TT	5	CT	21	CC	22	48
19	rs2268641	AA	4	AG	21	GG	22	47
20	rs2268640	CC	3	TC	18	TT	27	48
21	rs2268639	TT	3	TA	22	AA	22	48
22	rs2206942	AA	6	AG	24	GG	18	48
23	rs2894420	AA	9	AG	27	GG	11	47
26	rs4714211	GG	7	AG	27	AA	13	47
27	rs10305525	AA	0	CA	9	CC	39	48
28	rs9296291	CC	3	TC	17	TT	28	48
29	rs9968886	AA	0	GA	12	GG	36	48
30	rs2143733	GG	7	GT	27	TT	14	48
31	rs9296292	CC	4	CT	20	TT	24	48

### Gastric Emptying Rate

Mean ± SD gastric emptying rate for all participants was 68 ± 16 and 41 ± 8 min for T_½_ and T_lag_, respectively. Median (quartiles) gastric emptying rate for all participants was 63 (55–78) and 40 (36–44) min for T_½_ and T_lag_, respectively.

#### By Genotype

Results for T_lag_ and T_½_ according to genotype are shown in **Tables 3**, **4**, respectively. Differences in median gastric emptying T_lag_ were seen for SNP 10 rs742764 and SNP 11 rs2254336. For SNP 10 rs742764, gastric emptying T_lag_ was faster in genotype CC compared to genotype TT (*P =* 0.006) and TC (*P =* 0.006) by 15%. T_½_ was also tending to significance with differences of 18% (*P =* 0.061). For SNP 11 rs2254336, gastric emptying T_lag_ was slower in genotype AA compared to genotype TT (*P =* 0.04) and TA (*P =* 0.036) by 19 and 10%, respectively. T_½_ showed a slight trend to be slower than both other groups by 20 and 15% but this did not reach statistical significance (*P =* 0.138). No significant differences in gastric emptying rate were seen between genotypes for all other SNPs, although T_½_ tended toward significance for SNP 9 rs9283907 (*P =* 0.054) and T_lag_ tended to significance for SNP 5 rs2268657 (*P =* 0.087) and SNP 15 rs7766663 (*P =* 0.076).

**Table 3:** Gastric emptying T_lag_ results according to genotype.

	SNP ID	Homozygous minor allele			Heterozygous			Homozygous major allele			*P*-value
		Genotype	Median	Quartiles	Genotype	Median	Quartiles	Genotype	Median	Quartiles	
1	rs7738586	–	–	–	CA	37	34–41	CC	40	37–45	0.274
2	rs9380825	AA	42	61–80	AG	41	37–45	GG	37	36–40	0.349
4	rs926674	TT	37	37–41	TC	38	36–40	CC	41	36–46	0.448
5	rs2268657	GG	42	37–44	AG	38	37–46	AA	37	34–39	0.087
6	rs13202369	GG	34	31–36	AG	41	37–49	AA	39	36–42	0.295
7	rs3799707	TT	42	36–43	GT	40	35–43	GG	40	37–45	0.696
8	rs10305432	CC	36	35–37	CT	41	39–43	TT	40	35–46	0.267
9	rs9283907	AA	29	29–29	AG	37	35–41	GG	41	37–44	0.158
10	rs742764	CC	35^∗^	30–36	TC	41	39–45	TT	41	37–46	**0.008**
11	rs2254336	TT	36	34–41	TA	39	35–42	AA	43†	39–49	**0.031**
12	rs910163	CC	37	37–39	TC	40	36–45	TT	41	36–44	0.788
14	rs6923761	AA	43	41–48	GA	40	37–43	GG	37	34–41	0.222
15	rs7766663	GG	36	31–40	GT	40	38–44	TT	42	37–48	0.076
16	rs932443	GG	37	36–41	AG	40	38–45	AA	41	35–44	0.634
17	rs2268646	–	–	–	AG	40	37–48	GG	40	35–43	0.493
18	rs2300614	TT	36	35–37	CT	40	37–44	CC	42	36–46	0.210
19	rs2268641	AA	37	37–38	AG	40	35–44	GG	41	35–44	0.724
20	rs2268640	CC	37	37–39	TC	40	34–45	TT	41	37–44	0.741
21	rs2268639	TT	37	37–39	TA	40	35–44	AA	41	36–44	0.816
22	rs2206942	AA	39	37–51	AG	40	36–43	GG	41	32–46	0.878
23	rs2894420	AA	43	39–49	AG	39	36–42	GG	37	36–48	0.470
26	rs4714211	GG	36	33–39	AG	40	37–45	AA	41	37–44	0.244
27	rs10305525	–	–	–	CA	37	36–41	CC	40	36–45	0.369
28	rs9296291	CC	37	37–39	TC	40	35–45	TT	41	37–43	0.812
29	rs9968886	–	–	–	GA	37	36–45	GG	41	36–43	0.543
30	rs2143733	GG	37	37–48	GT	39	35–43	TT	42	39–48	0.549
31	rs9296292	CC	37	34–38	CT	39	34–44	TT	41	38–45	0.166

*Values are minutes. ^∗^Significantly faster than other two genotypes (*P* < 0.01). †Significantly slower than other two genotypes (*P* < 0.05). Bold values indicate significant difference*.

**Table 4:** Gastric emptying T_½_ results according to genotype.

	SNP ID	Homozygous minor allele			Heterozygous			Homozygous major allele			*P*-value
		Genotype	Median	Quartiles	Genotype	Median	Quartiles	Genotype	Median	Quartiles	
1	rs7738586	–	–	–	CA	62	56–72	CC	64	55–79	0.919
2	rs9380825	AA	71	61–80	AG	64	56–80	GG	60	55–70	0.650
4	rs926674	TT	59	56–62	TC	60	55–71	CC	64	56–82	0.569
5	rs2268657	GG	72	59–83	AG	61	56–73	AA	59	53-67	0.203
6	rs13202369	GG	70	58–81	AG	62	54–77	AA	64	56–73	0.935
7	rs3799707	TT	61	57–66	GT	59	54–83	GG	66	59–78	0.452
8	rs10305432	CC	59	56–71	CT	65	57–75	TT	62	55–80	0.852
9	rs9283907	AA	41	41–41	AG	56	54–62	GG	67	59–82	0.054
10	rs742764	CC	54	53–59	TC	66	56–80	TT	66	59–81	0.061
11	rs2254336	TT	59	53–68	TA	62	54–75	AA	71	62–85	0.138
12	rs910163	CC	59	56–60	TC	66	54–82	TT	62	57–73	0.431
14	rs6923761	AA	72	66–81	GA	62	56–74	GG	59	54–83	0.297
15	rs7766663	GG	57	53–66	GT	62	54–79	TT	71	61–81	0.168
16	rs932443	GG	60	59–68	AG	65	54–83	AA	62	57–72	0.820
17	rs2268646	–	–	–	AG	67	57–87	GG	63	54–76	0.485
18	rs2300614	TT	54	53–59	CT	65	54–83	CC	66	58–73	0.120
19	rs2268641	AA	57	54–59	AG	65	54–80	GG	63	58–73	0.283
20	rs2268640	CC	59	56–60	TC	66	54–82	TT	62	57–73	0.453
21	rs2268639	TT	59	56–60	TA	65	55–79	AA	63	56–73	0.475
22	rs2206942	AA	60	57–86	AG	65	55–78	GG	63	55–73	0.959
23	rs2894420	AA	72	64–92	AG	62	55–72	GG	59	54–81	0.187
26	rs4714211	GG	54	54–60	AG	64	56–75	AA	68	59–89	0.233
27	rs10305525	–	–	–	CA	59	55–68	CC	64	55–79	0.412
28	rs9296291	CC	59	56–60	TC	66	54–83	TT	62	56–72	0.386
29	rs9968886	–	–	–	GA	61	56–92	GG	65	54–74	0.934
30	rs2143733	GG	59	55–77	GT	64	55–79	TT	65	59–73	0.791
31	rs9296292	CC	56	52–59	CT	65	54–81	TT	66	60–77	0.125

*Values are minutes*.

#### By Phenotype

Results for T_lag_ and T_½_ according to phenotype are shown in **Tables 5**, **6**, respectively. A significant effect of the minor allele on median gastric emptying T_½_ was seen for SNP 9 rs9283907. Participants with one or two A alleles had faster T_½_ than participants homozygous for the allele G by 18% (*P* = 0.033). A significant effect of the minor allele on median gastric emptying T_lag_ was also seen for SNP 5 rs2268657 and SNP 11 rs2254336. Participants with one or two G alleles had slower T_lag_ than participants homozygous for the allele A by 11% (*P* = 0.028), and participants with one or two T alleles had faster T_lag_ than participants homozygous for the allele A by 13% (*P* = 0.012), respectively. No differences in gastric emptying rate were seen between phenotypes for all other SNPs, though T_½_ tended toward significance for SNP 11 rs2254336 (*P* = 0.055) and SNP 15 rs7766663 (*P* = 0.097).

**Table 5:** Gastric emptying T_lag_ results according to phenotype.

	SNP ID	Homozygous major allele				Heterozygous/homozygous minor allele				*P*-value
		Phenotype	*n*	Median	Quartiles	Phenotype	*n*	Median	Quartiles	
1	rs7738586	CC	38	40	37–45	A	10	37	34–41	0.274
2	rs9380825	GG	15	37	36–40	A	33	41	37–44	0.157
4	rs926674	CC	39	41	36–46	T	9	37	36–40	0.239
5	rs2268657	AA	11	37^∗^	34–39	G	36	41	37–45	**0.028**
6	rs13202369	AA	25	39	36–42	G	23	41	36–48	0.627
7	rs3799707	GG	26	40	37–45	T	22	40	34–43	0.396
8	rs10305432	TT	26	40	35–46	C	22	41	37–42	0.844
9	rs9283907	GG	38	41	37–44	A	10	36	33–41	0.137
10	rs742764	TT	14	41	37–46	C	33	40	35–43	0.492
11	rs2254336	AA	16	43	39–49	T	32	39^∗^	34–41	**0.012**
12	rs910163	TT	27	41	36–44	C	21	39	36–44	0.950
14	rs6923761	GG	13	37	34–41	A	35	40	37–44	0.197
15	rs7766663	TT	15	42	37–48	G	33	39	35–42	0.136
16	rs932443	AA	22	41	35–44	G	26	40	36–44	0.983
17	rs2268646	GG	38	40	35–43	A	10	40	37–48	0.493
18	rs2300614	CC	22	42	36–46	T	26	39	35–42	0.330
19	rs2268641	GG	22	41	35–44	A	25	39	36–42	0.856
20	rs2268640	TT	27	41	37–44	C	21	39	35–44	0.546
21	rs2268639	AA	22	41	36–44	T	25	39	35–42	0.701
22	rs2206942	GG	18	41	32–46	A	30	40	36–44	0.806
23	rs2894420	GG	11	37	36–48	A	36	40	36–43	0.930
26	rs4714211	AA	13	41	37–44	G	34	40	35–44	0.497
27	rs10305525	CC	39	40	36–45	A	9	37	36–41	0.369
28	rs9296291	TT	28	41	37–43	C	20	40	35–44	0.908
29	rs9968886	GG	36	41	36–43	A	12	37	36–45	0.543
30	rs2143733	TT	14	42	39–48	G	34	39	35–44	0.276
31	rs9296292	TT	24	41	38–45	C	24	38	34–43	0.107

*Values are minutes. ^∗^Significantly faster emptying rate compared to other phenotype (*P* < 0.05). Bold values indicate significant difference*.

**Table 6:** Gastric emptying T_½_ results according to phenotype.

	SNP ID	Phenotype	*n*	Median	Quartiles	Phenotype	*n*	Median	Quartiles	*P*-value
1	rs7738586	CC	38	64	55–79	A	10	62	56–72	0.919
2	rs9380825	GG	15	60	55–70	A	33	64	56–80	0.367
4	rs926674	CC	39	64	56–82	T	9	59	54–65	0.355
5	rs2268657	AA	11	59	53–67	G	36	64	56–78	0.119
6	rs13202369	AA	25	64	56–73	G	23	62	54–80	0.757
7	rs3799707	GG	26	66	59–78	T	22	60	54–76	0.218
8	rs10305432	TT	26	62	55–80	C	22	65	55–76	0.836
9	rs9283907	GG	38	67	59–82	A	10	55^∗^	53–61	**0.033**
10	rs742764	TT	14	66	59–81	C	33	62	54–77	0.478
11	rs2254336	AA	16	71	62–85	T	32	60	54–74	0.055
12	rs910163	TT	27	62	57–73	C	21	64	54–80	0.950
14	rs6923761	GG	13	59	54–83	A	35	64	57–75	0.516
15	rs7766663	TT	15	71	61–81	G	33	61	54–77	0.097
16	rs932443	AA	22	62	57–72	G	26	65	54–82	0.702
17	rs2268646	GG	38	63	54–76	A	10	67	57–87	0.485
18	rs2300614	CC	22	66	58–73	T	26	62	54–79	0.521
19	rs2268641	GG	22	63	58–73	A	25	62	54–77	0.733
20	rs2268640	TT	27	62	57–73	C	21	64	54–80	0.755
21	rs2268639	AA	22	65	55–79	T	25	62	54–77	0.806
22	rs2206942	GG	18	63	55–73	A	30	63	55–79	0.774
23	rs2894420	GG	11	59	54–81	A	36	64	56–74	0.715
26	rs4714211	AA	13	68	59–89	G	34	62	54–73	0.385
27	rs10305525	CC	39	64	55–79	A	9	59	55–68	0.412
28	rs9296291	TT	28	62	56–72	C	20	65	54–81	0.875
29	rs9968886	GG	36	65	54–74	A	12	61	56–92	0.934
30	rs2143733	TT	14	65	59–73	G	34	63	54–79	0.691
31	rs9296292	TT	24	66	60–77	C	24	60	54–78	0.239

*Values are minutes. ^∗^Significantly faster emptying rate compared to other phenotype (*P* < 0.05). Bold values indicate significant difference*.

### Body Mass Index and Body Fat Percentage

Median (quartiles) BMI for all participants was 23.1 (21.6–25.3) kg.m^-2^. No effect of genotype on BMI was found for all SNPs although SNP 15 rs7766663 tended to significance [GG, 24.5 (21.6–26.2) kg.m^-2^ vs. GT, 24.0 (22.2–26.0) kg.m^-2^ vs. TT, 22.1 (21.1–23.3) kg.m^-2^; *P =* 0.091]. Analysis by phenotype, however, revealed median BMI was significantly higher in participants with one or two minor alleles compared to homozygotes of the major allele for SNP 12 rs910163 [TT, 22.2 (21.3–24.2) kg.m^-2^ vs. CC/CT, 24.6 (22.6–26.7) kg.m^-2^; *P =* 0.039] and SNP 15 rs7766663 [TT, 22.1 (21.1–23.3) kg.m^-2^ vs. GG/GT, 24.0 (22.1–26.3) kg.m^-2^; *P =* 0.028].

Median (quartiles) body fat for all participants was 18.3 (14.1–22.2)%. No effect of genotype nor phenotype was seen for any SNPs, although SNP 28 rs9296291 tended to significance for phenotype [TT, 17.2 (13.5–19.7)% vs. CC/CT, 20.8 (15.5–24.9)%; *P =* 0.061].

## Discussion

The results of this study showed several Tag SNPs of the GLP1R gene are significantly associated with the gastric emptying rate of a glucose solution in healthy young Caucasian men. Two neighboring polymorphisms, SNPs 10 rs742764 and 11 rs2254336, were found to be significantly associated with gastric emptying rate by genotype. In addition, SNPs 5 rs2268657, 9 rs9283907, and 15 rs7766663 tended to significance. Furthermore, three SNPs, 5 rs2268657, 9 rs9283907, and 11 rs2254336, were found to be significantly associated with gastric emptying rate by phenotype. SNP 15 rs7766663 also tended to significance. Thus, significant associations between genetic variation and gastric emptying rate were found for four Tag SNPs by one or more measures of genetic association. Tag SNP 5 rs2268657 is situated in intron one and the other three 9, 10, 11 are situated in intron 3.

The aforementioned Tag SNPs identified to be associated with gastric emptying rate signify a region where a causative variant is most likely to reside (Xia and Grant, 2013). As three of the associated SNPs are neighboring Tag SNPs, this presents a genomic region that warrants further investigation with particularly high interest. Approximately 3000 bp exist between the locations of SNP 9 rs9283907 and 10 rs742764 and similarly between SNP 10 rs742764 and 11 rs2254336. Spanning this whole 6132 bp “hot spot” region, a total of 129 SNPs have been sequenced to date. No known functional SNPs within the GLP1R gene have currently been identified within this particular region or within introns 1, 3, or 5. However, it is widely known that SNPs in regulatory elements residing within intronic regions can alter silencing, enhancer, or splicing events (Chorev and Carmel, 2012; Lee et al., 2012). Further work by *in silico* analysis or multi-array analysis of all 129 known SNPs within this region to narrow down on the precise SNP or SNPs responsible for the observed differences in gastric emptying rate should therefore be conducted. The genetic variants surrounding SNP 5 rs2268657 in intron one should also be further investigated as SNPs within intron one of several genes have been shown to influence gene transcription events (Murani et al., 2009; Berulava and Horsthemke, 2010).

Variants in the proximity of SNP 15 rs7766663 also provide a directive area of further research in gastric emptying regulation as it tended toward significance but was also significantly associated with BMI by phenotype. This association may signal a link between gastric emptying rate and BMI but further participants are required to confirm these concurrent associations. Indeed, further potential “links” or associations may also be identified with a much larger sample size which will increase the power of future studies.

The two additional missense SNPs in close proximity to the locus of the variant seen in the mice model by Kumar et al. (2008) did not show any variants in the sample population of this present study. It may be that the variant is rare or non-existent in the Caucasian population. The MAFs for these two missense SNPs are unknown. The other additional missense SNP selected for its previous association with insulin secretion (Sathananthan et al., 2010) also showed no variants in this participant group. This was mostly likely due to its small MAF of 0.0646 indicating the SNP is somewhat rare. Tag SNPs 6 rs13202369 and 14 rs6923761, located in intron 1 and exon 5, respectively, have previously been reported to alter insulin secretion responses to intravenous GLP-1 (Vella et al., 2009; Sathananthan et al., 2010). These were not found to be associated with gastric emptying rate in this study, however.

## Conclusion

The results of this targeted gene study to investigate the potential influence of GLP1R genetic variation on gastric emptying rate in humans revealed several Tag SNPs to be associated with gastric emptying rate of a glucose solution in healthy Caucasian men. This suggests that genetic variation within the GLP1R gene may influence gastric emptying rate in humans. Further work should be undertaken to identify the precise SNP or SNPs responsible and functional analysis conducted. In addition, this experiment should be replicated with the use of foods more representative of a typical meal. Furthermore, this association study should be repeated with a larger population sample to independently confirm the detected associations between GLP1R genetic variation and gastric emptying rate.

## Author Contributions

Experimental trials and DNA extractions were performed at Manchester Metropolitan University. All other genotyping work was performed at CIGMR in Manchester University. AY, GE, and JA conceived and designed the experiments. AY and JA performed the experiments. AY analyzed the data and wrote the paper with contributions from GE, JA, JM, RM, and WG. All authors read and approved the final manuscript and agreed to be accountable for all aspects of the work in ensuring that questions related to the accuracy or integrity of any part of the work are appropriately investigated and resolved.

## Conflict of Interest Statement

The authors declare that the research was conducted in the absence of any commercial or financial relationships that could be construed as a potential conflict of interest.

## Footnotes

1 MyProtein.com

2 www.hapmap.org
